# Surgical teaching in urology: patient safety and educational value of ‘LIVE’ and ‘SEMI-LIVE’ surgical demonstrations

**DOI:** 10.1007/s00345-018-2291-x

**Published:** 2018-04-21

**Authors:** Jaap D. Legemate, Stefano P. Zanetti, Jan Erik Freund, Joyce Baard, Jean J. M. C. H. de la Rosette

**Affiliations:** 1 Department of Urology, AMC University Hospital, Meibergdreef 9, 1105 AZ Amsterdam Z-O, The Netherlands; 2 Department of Urology, Fondazione IRCCS Ca’ Granda Ospedale Maggiore Policlinico, University of Milan, Milan, Italy

**Keywords:** Live surgery, Semi-live surgery, Educational value, Safety

## Abstract

**Purpose:**

To evaluate the opinion of urologists and their audience regarding patient safety and educational value of live surgical demonstrations (LSD) and semi-live surgical demonstrations (semi-LSD).

**Methods:**

Following the ‘2017 Challenges in Endourology’ meeting, a survey addressing patient safety and the educational value of LSD and semi-LSD was disseminated online to all participants. Survey outcomes of LSD and semi-LSD were compared.

**Results:**

All 279 respondents attended both LSD and semi-LSD. Overall, 53% of said respondents stated that patient safety was always the highest priority for LSD, while 74% noted the same for semi-LSD. The complication risk in LSD was perceived equal by 57% of the respondents when compared to cases of similar difficulty in routine practice, while 38% perceived it as a greater risk. For semi-LSD, the complication risk was perceived equal by 84%, while 5% perceived it to be a greater risk in comparison to general practice. On a scale from 0 (no value) to 10 (highly valuable), the average educational value of LSD and semi-LSD was rated 8.4 and 8.3, respectively. A substantial percentage of the surgeons who perform LSD express concerns that live surgery is not the optimal setting to ensure patient safety.

**Conclusions:**

LSD remains a popular tool for surgical education among urologists and their audience. However, patient safety remains a concern and is perceived less of a concern for semi-LSD. The educational value of LSD and semi-LSD was scored equally high. Therefore, we should consider to advocate the use of semi-LSD more often.

**Electronic supplementary material:**

The online version of this article (10.1007/s00345-018-2291-x) contains supplementary material, which is available to authorized users.

## Introduction

Live surgical demonstrations (LSD) are a popular educational tool in the field of urology. Technical innovations facilitate high-quality broadcasts for large audiences. The increase of LSD and their popularity fuelled the debate on patient safety and their educational value [1–6].

Pre-recorded videos of surgical demonstrations with post hoc moderation, referred to as semi-live surgical demonstrations (semi-LSD), are an alternative to LSD and might overcome the controversy around patient safety. Previous surveys revealed a positive perception of the educational value of semi-LSD [7, 8]. Nonetheless, the attractiveness of semi-LSD is often challenged by bias of pre-selection [9]. The present study evaluates the perception on safety and educational value of both the audience and the performing urologists directly after two consecutive days of LSD and semi-LSD. The results of this study may help us to determine the current position of semi-LSD to LSD. This will help us to establish surgical education that is in line with the requirements for patient safety and educational needs.

## Materials and methods

### Study aims

To evaluate the opinion of congress participants and faculty members on patient safety and educational value of LSD compared to semi-LSD.

### Organisation

In the plenary hall of the congress ‘Challenges in Endourology 2017’ (CIE2017) in Paris, participants could attend streamed LSD and semi-LSD sessions on two consecutive days. A total of 24 surgical procedures were demonstrated (8 LSD and 16 semi-LSD). LSD were performed at Tenon Hospital in Paris with live streaming and moderation in the plenary hall. LSD and semi-LSD included transurethral resections of bladder tumors (TURBT), transurethral desobstruction of the prostate, retrograde intrarenal surgeries (RIRS), and percutaneous nephrolithotomies (PNL). All LSD and semi-LSD were performed by specialized endourologists with experience in LSD. French legislation allowed only endourologists with French nationality to perform LSD. Independent urologists were allocated to advocate patient safety. They were present in the operation room during all LSD.

Semi-LSD mostly consisted of unedited video recordings of surgical procedures performed at the surgeons’ home institution. During plenary sessions, these videos were shown and discussed by the performing surgeon and moderators.

Before and during the CIE2017, the survey for this study was brought to attention. After concluding the CIE2017 meeting, all participants were invited via email to fill out a survey. Google Documents Software was used for this anonymous online survey. Participants had to complete the survey in order to receive their certificate of attendance including CME points. One reminder was sent.

### Survey and analyses

The items addressed in the survey were based on the literature and on input from the organizers, who coordinated the live and semi-live surgeries [7, 9–12]. A committee of ten test participants reviewed the survey questions in several rounds to ensure the comprehensibility. The survey was finalized by the congress organizers who are experienced in conducting surveys at meetings. All questions contained fixed reply options. The first part of the survey consisted of questions on participant baseline characteristics, the second and the third part concerned patient safety and educational value, respectively. The fourth part was reserved for CIE2017 faculty members and evaluated their experience and preferences in providing LSD and semi-LSD. All outcomes were presented as descriptive data. Analyses were performed using SPSS statistics 24.0. A subgroup analysis was performed based on whether respondents had ever performed LSD themselves. Respondents who did not attend any LSD and semi-LSD were excluded from the analyses. The complete survey can be found in the supplementary materials.

## Results

### Participants

Four hundred and eighty-five participants (including 87 faculty members) from 64 countries worldwide attended the CIE2017. In total, 282 (58%) participants completed the survey. Respondents’ characteristics and data about experience with LSD and semi-LSD are presented in Table 1. Prior to the congress, 93% (263/282) and 77% (217/282) of the respondents had experienced LSD and semi-LSD, respectively. After the CIE2017, 279 of the 282 respondents had attended both LSD and semi-LSD. Three respondents were excluded due to lack of experience with LSD. During the CIE2017 meeting, 90% of the respondents attended RIRS-, 81% TURBT-, 84% transurethral prostate desobstructions and 71% PNL-demonstrations. Fifty-one percent of the respondents (143/279) had previously performed LSD themselves and 39% (108/279) had performed semi-LSD.

**Table 1:** Participants characteristics

Description	No. of respondents (*n* = 279) *n*. (%)
Age group (years)	
≤ 30	19 (6.8)
31–50	171 (61.3)
51–70	89 (31.9)
Profession	
Urologist	234 (83.9)
Resident	30 (10.7)
Industry	8 (2.9)
Nurse	2 (0.7)
Other	5 (1.8)
Number of live surgeries attended before CIE2017	
0	16 (5.7)
0–15	141 (50.6)
> 15	122 (43.7)
Number of semi-LSD attended before CIE2017	
0	62 (22.2)
0–15	164 (58.8)
> 15	53 (19.0)
Number of LSD performed by the attendee	
0	136 (48.7)
0–15	114 (40.9)
> 15	29 (10.4)
Number of semi-LSD performed by the attendee	
0	171 (61.3)
0–15	88 (31.5)
> 15	20 (7.2)

### Faculty

Prior to the CIE2017 meeting 79% of the faculty members (69/87) had performed LSD at their home institution and 75% (65/87) at foreign institutions. When providing surgical education, 40% of the faculty members indicated to have a preference for LSD, 21% for semi-LSD and 39% had no preference. Twenty percent (14/69) of the faculty members that had previously performed LSD at their home institution stated that pressure had affected their performance almost always or often while 61% (42/69) stated that it rarely did. Of the sixty-five faculty members that had performed LSD at a foreign institution, 26% (17/65) stated that pressure had affected their performance almost always or often while 60% (39/65) stated it rarely did. Eleven faculty members (13%) noted that a jet lag had almost always or often negatively affected their performance during LSD. Next, 33% of all faculty members (29/87) believed that unfamiliarity with surgical staff or equipment often or almost always negatively affects the surgical performance during LSD.

### Safety

The data on patient safety for all respondents are presented in Table 2 and for the subgroup of participants who performed LSD themselves in Table 3. Fifteen percent of all respondents (41/279) stated that patient safety was often or almost always not the highest priority during LSD. For semi-LSD, 8% of all respondents (21/279) felt that patient safety was often or almost always not the highest priority. In the subgroup analysis, 18% of the LSD performers (26/143) had the impression that patient safety was often or almost always not the highest priority.

**Table 2:** Outcomes of survey questions on safety and educational value of LSD and semi-LSD for all respondents

Question	LSD (*n* = 279)	SEMI-LSD (*n* = 279)
Were you concerned that the patients’ safety was NOT the highest priority?		
Never or rarely	238 (85.3)	258 (92.5)
Often or almost always	41 (14.7)	21 (7.5)
Were you concerned that the patients’ outcomes may have been compromised?		
Never or rarely	240 (86.0)	263 (94.3)
Often or almost always	39 (14.0)	16 (5.7)
Do you think that the complication risk is higher, equal or lower as compared to routine practice?		
Higher	105 (37.6)	15 (5.4)
Equal	160 (57.4)	235 (84.2)
Lower	14 (5.0)	29 (10.4)
Did you have the impression that there were factors (pressure and anxiety) influencing the surgeons’ performance?		
Never or rarely	179 (64.2)	254 (91.0)
Often or almost always	100 (35.8)	25 (9.0)
How would you rate the overall educational value?		
1–10 (1 = poor) mean (SD)	8.4 (1.3)	8.3 (1.6)

**Table 3:** Outcomes of participants who have performed LSD themselves and outcomes of who did not perform LSD themselves

Question	Not performed LSD *n* = 136 (48.7%)	Performed LSD *n* = 143 (51.3%)
How many times have you performed LIVE surgeries yourself?		
< 5	0 (0)	85 (59.4)
> 5	0 (0)	58 (40.6)
During LSD, were you concerned that the patients’ safety was NOT the highest priority?		
Never or rarely	121 (89.0)	117 (81.8)
Often or almost always	15 (11.0)	26 (18.2)
During semi-LSD, were you concerned that the patients’ safety was NOT the highest priority?		
Never or rarely	127 (93.4)	131 (91.6)
Often or almost always	9 (6.6)	12 (8.4)
During LSD, were you concerned that the patients’ outcomes may have been compromised?		
Never or rarely	123 (90.4)	117 (81.8)
Often or almost always	13 (9.6)	26 (18.2)
During semi-LSD, were you concerned that the patients’ outcomes may have been compromised?		
Never or rarely	133 (97.8)	130 (90.9)
Often or almost always	3 (2.2)	13 (9.1)
During LSD surgeries, did you have the impression that there were factors (pressure and anxiety) influencing the surgeons’ performance?		
Never or rarely	94 (69.1)	85 (59.4)
Often or almost always	42 (30.9)	58 (40.6)
During semi-LSD surgeries, did you have the impression that there were factors (pressure and anxiety) influencing the surgeons’ performance?		
Never or rarely	130 (95.6)	124 (86.7)
Often or almost always	6 (4.4)	19 (13.3)
Do you think that the complication risk is higher, equal or lower during LSD surgery, as compared to routine practice?		
Higher	50 (36.8)	55 (38.5)
Equal	80 (58.8)	80 (55.9)
Lower	6 (4.4)	8 (5.6)
Do you think that the complication risk is higher, equal or lower during semi-LSD surgery, as compared to routine practice?		
Higher	4 (2.9)	11 (7.7)
Equal	116 (85.3)	119 (83.2)
Lower	16 (11.8)	13 (9.1)
How would you rate the overall educational value of LSD surgery?		
1–10 (1 = poor) mean (SD)	8.6 (1.3)	8.3 (1.4)
How would you rate the overall educational value of semi-LSD surgery?		
1–10 (1 = poor) mean (SD)	8.6 (1.3)	7.9 (1.8)
Number of respondents that would participate less often in surgical education if SEMI-LIVE surgery would replace LIVE surgery?	32 (23.5)	60 (42.0)

In general, respondents felt that treatment outcomes are more often compromised after LSD than after semi-LSD. The complication risk of LSD in comparison to routine practice of equal difficulty was perceived as higher by 38% of both all respondents (105/279) and the LSD performers (55/143). For semi-LSD, the complication risk in comparison to routine practice of equal difficulty was judged as higher by 5% of all respondents (15/279) and 8% of the LSD performers (11/143).

For LSD, 36% of all respondents (100/279) stated that the performance of the surgeon has been almost always or often influenced by stress and pressure. For semi-LSD, 9% of all respondents (25/279) felt that stress and pressure have almost always or often affected the surgeon’s performance.

For LSD, 23% of all respondents (63/279) felt that the audience has often or almost always distracted the surgeons, which may negatively affect the surgeon’s performance. Nevertheless, 94% of all respondents (263/279) agreed that the interaction between the audience and the performing surgeon is well organized for both LSD and semi-LSD.

### Educational value

The results on the educational value for all respondents are presented in Table 2 and for the subgroups in Table 3. A global overview of the pros and cons of LSD as compared to semi-LSD is presented in Table 4. LSD and semi-LSD were perceived as equally valuable in learning new tips and tricks and in learning how to manage complications. The overall educational value of LSD and semi-LSD was scored equally high by the respondents on a scale from 0 to 10 (10 = highly educational). On average, LSD performers rated the educational value for LSD as 8.3 and for semi-LSD as 7.9. The non-performers perceived the average educational value for both LSD and semi-LSD as 8.6. In total, 82% of all respondents would like to see more edited semi-live case discussions, while 33% of all respondents would attend less educational demonstrations if semi-LSD would replace LSD.

**Table 4:** Pros and cons of LSD versus semi-LSD

Item	
Concerns on patient safety	LSD > semi-LSD
Concerns on compromised outcomes	LSD > semi-LSD
Pressure and anxiety during surgery	LSD > semi-LSD
Distraction during the surgery by the audience	LSD > semi-LSD
Unfamiliarity with the environment	LSD > semi-LSD
Overall educational value	LSD = semi-LSD
Learn tips and tricks	LSD = semi-LSD
Learn to manage complications	LSD = semi-LSD
Attractiveness of the learning tool	LSD > semi-LSD

## Discussion

This study presents the current opinion on patient safety and educational value of LSD and semi-LSD of a large group of international congress attendees in the field of endourology. Patient safety is perceived as more of a concern for LSD than for semi-LSD. Moreover, a substantial percentage of surgeons who had performed LSD themselves expressed their concerns that live surgery may not be the ideal surgical setting to ensure patient safety. Even though the educational value of LSD and semi-LSD is perceived as equally high, one-third of the congress attendees would attend less surgical demonstrations if semi-LSD would replace LSD.

This survey was conducted among the faculty members and the attendees of the CIE2017 meeting. Considering that surgical demonstrations are used as an educational tool, the opinion of the audience on the educational value and patient safety is of great importance. However, as the audience may not have performed LSD or semi-LSD themselves, their opinion may give rise to subjective conclusions about patient safety. The opinion of the surgeons who perform LSD and semi-LSD themselves may be the most valuable with regard to patient safety. Notably, a high percentage of 51% of all survey respondents had performed LSD themselves. More than one-third of these LSD performers believed that the complication risk for LSD is higher than that for routine practice of equal difficulty. These concerns may be related to the fact that a substantial percentage of faculty members indicated that pressure, stress, jet lag or unfamiliarity with the OR-environment had negatively affected their performance during LSD.

These concerns are in concordance with the outcomes of other survey studies [7, 9–12]. In the study by Elsamra et al., 22% of the survey respondents had doubts about the ethical value of LSD and 41% of the respondents would not participate as a patient themselves [9]. Also Finch et al. identified that survey respondents were significantly less likely to participate in LSD as a patient or to recommend LSD to family and friends [7].

On the other hand, observational studies, comparing the outcomes of LSD with routine practice, give less cause for concern [6, 13, 14]. Brunckhorst et al. reviewed the literature from 1980 until 2014 on LSD outcomes, identifying eight studies in different medical fields. In three of these studies, the success rates of LSD were lower than of routine practice, yet there were no statistically significant differences in complication rates [13]. More recently, three studies evaluated the outcomes of live endourological procedures and live robot-assisted radical prostatectomies. These studies did not report statistically significant differences in outcomes in comparison to routine cases [6, 14, 15].

In this study, semi-LSD evoke less safety concerns than LSD. The educational value of semi-LSD and LSD was rated equally high. These findings are in line with the results of the survey study by Finch et al. among delegates at the British Association of Urological Surgeons. Finch concluded that semi-LSD offer similar educational opportunities as LSD, but with potential benefits for both the patient and the surgeon [7]. Considering these findings, the controversy about patient safety may be resolved if semi-LSD would replace LSD. However, as our study identified, one-third of the respondents would attend less surgical demonstrations if semi-LSD will replace LSD. Since the educational value of semi-LSD and LSD is perceived as equally high, it may seem that this potential loss of audience is caused by a lower attractiveness of semi-LSD. One might argue that the additional attractiveness of LSD is comparable to watching live sports, where the excitement of spectatorship partially arises from the unpredictability of events and the uncertainty of outcomes. Though the high level evidence comparing LSD and semi-LSD are lacking, the data we present here suggest that surgeons should consider using semi-LSD in place of LSD.

### Limitations

In survey studies, there is a substantial risk of response bias. The respondents could feel more attracted to the topic than non-respondents, which may influence outcomes. Next, the survey study only reflects the opinion of respondents, which may result in subjectivity of outcomes. At last, with disregard to prior LSD experience, all respondents were exposed to LSD by French surgeons at the CIE2017 only. Thus, we may assume that there were no language barriers with the surgical staff and that the majority of the LSD performers were familiar with the setting and equipment. This may have had a positive effect on safety but it may not fully represent the setting of live surgical events with visiting foreign surgeons.

## Conclusion

Live surgical demonstrations remain a popular educational tool but patient safety is still a concern. Not only the audience but also a substantial percentage of the surgeons who perform LSD themselves are concerned that live surgery is not the optimal setting to ensure patient safety. The educational value of semi-LSD is perceived as equally high as for LSD, but with potential benefits for patients and surgeons. Therefore, we should face our concerns and consider to shift towards semi-LSD for surgical education. To reach such a shift, the attractiveness of semi-LSD should be promoted.

## Electronic supplementary material

Below is the link to the electronic supplementary material.

Supplementary material 1 (DOC 34 kb)

## Abbreviations

LSD: Live surgical demonstration
Semi-LSD: Semi-live surgical demonstration
