# Supplementary material for: Surgical teaching in urology: patient safety and educational value of ‘LIVE’ and ‘SEMI-LIVE’ surgical demonstrations
Source: World J Urol. 2018 Apr 21;36(10):1673–9. doi: 10.1007/s00345-018-2291-x (PMC6153636; doi:10.1007/s00345-018-2291-x)
Supplement: Supplementary file 1 — Supplementary material 1 (DOC 34 kb) [file 345_2018_2291_MOESM1_ESM.doc]

**Title: Surgical teaching in urology: patient safety and educational value of ‘LIVE’ and ‘SEMI-LIVE surgical demonstrations’.**

Authors: Jaap D Legemate*^a^, Stefano P Zanetti^b^, Jan Erik Freund^a^, Joyce Baard, Jean JMCH de la Rosette^a^

^a^Department of Urology, AMC University Hospital, Amsterdam, the Netherlands

^b^Department of Urology, Fondazione IRCCS Ca’ Granda Ospedale Maggiore Policlinico, University of Milan, Italy

*Corresponding author:

Jaap D Legemate, M.D.

Department of Urology

AMC University Hospital

Meibergdreef 9

1105 AZ Amsterdam Z-O

The Netherlands

Tel: +31-20-5664377

Fax: +31-20-5669585

E-mail: [j.d.legemate@amc.uva.nl](mailto:j.d.legemate@amc.uva.nl)

ORCID iD: 0000-0002-0517-4515

Key words: live surgery, semi-live surgery, educational value, safety

**Supplementary material: Survey live- and semi-live surgical demonstrations Challenges in Endourology 2017**

**Item 1: Participants characteristics**

1. **Are you participating as a faculty member in the meeting?**

yes/no

1. **Please indicate your age group.**

≤30/31-40/41-50/51-60/61-70/>70

1. **Which of the following options best describes your profession?**

urologist/resident/nurse/industry/other

1. **How many LIVE surgery events have you previously attended?**

0/<5/5-15/15-25/>25

1. **How many times have you performed LIVE surgeries yourself?**

0/<5/5-15/15-25/>25

1. **How many SEMI-LIVE surgery events have you previously attended?**

0/<5/5-15/15-25/>25

1. **How many times have you provided SEMI-LIVE surgical demonstrations yourself?**

0/<5/5-15/15-25/>25

1. **Which surgical demonstration sessions did you attend during the CIE 2017 Meeting?**

RIRS/TURBT/TURP/PNL/none

**Item 2: Patient’s safety**

1. **During LIVE surgeries, were you concerned that the patient’s safety was NOT the highest priority?**

never/rarely/often/almost always

1. **During SEMI-LIVE surgeries, were you concerned that the patient’s safety was NOT the highest priority?**

never/rarely/often/almost always

1. **During LIVE surgeries, were you concerned that the patient’s outcomes may have been compromised?**

never/rarely/often/almost always

1. **During SEMI-LIVE surgeries, were you concerned that the patient’s outcomes may have been compromised?**

never/rarely/often/almost always

1. **During LIVE surgeries, did you have the impression that there were factors (e.g. pressure and anxiety) influencing the surgeons’ performance?**

never/rarely/often/almost always

1. **During SEMI-LIVE surgeries, did you have the impression that there were factors (e.g. pressure and anxiety) influencing the surgeons’ performance?**

never/rarely/often/almost always

1. **Do you think that complications’ risk is higher, lower or equal during LIVE surgery, as compared to routine practice, taking into consideration the case is equal in level of difficulty?**

higher/equal/lower

1. **Do you think that complications risk is higher, lower or equal during SEMI-LIVE surgery, as compared to routine practice, taking into consideration the case is equal in level of difficulty?**

higher/equal/lower

1. **Did you ever have the impression that the surgeon was distracted by the audience, which negatively affected his/her surgical performance during a LIVE surgery?**

never/rarely/often/almost always

**Item 3: Educational value**

1. **I have learned new tips and tricks during LIVE surgery.**

strongly agree/agree/disagree/strongly disagree

1. **I have learned new tips and tricks during SEMI-LIVE surgery.**

strongly agree/agree/disagree/strongly disagree

1. **LIVE surgeries have helped me learn how to manage complications.**

strongly agree/agree/disagree/strongly disagree

1. **SEMI-LIVE surgeries have helped me learn how to manage complications.**

strongly agree/agree/disagree/strongly disagree

1. **Interaction between the surgeon and the observers was well organized during LIVE surgery.**

strongly agree/agree/disagree/strongly disagree

1. **Interaction between the surgeon and the observers was well organized during SEMI-LIVE surgery.**

strongly agree/agree/disagree/strongly disagree

1. **Would you participate less often in surgical education if LIVE surgery was replaced by SEMI-LIVE surgery?**

yes/no

1. **How would you rate the overall educational value of LIVE surgery?**

1-10 (1=poor)

**26) How would you rate the overall educational value of SEMI-LIVE surgery?**

1-10 (1=poor)

1. **How would you rate the educational value of case presentations during the "Nightmare Session" (Monday, May 22nd @ 18:30-19:30)?**

1-10 (1=poor)

1. **Would you like more case discussions?**

yes/no

**Item 4: Faculty members questionnaire**

**When providing surgical education, which modality do you prefer: SEMI-LIVE or LIVE surgery?**

live-surgery/equal preference/semi-live surgery

**Does stress or a pressure feeling negatively affect your performance when performing LIVE surgery at your home institution?**

never/rarely/often/almost always/ I have never performed LIVE surgery in my home institution

**Does stress or a pressure feeling negatively affect your performance when performing LIVE surgery as a visiting surgeon in a foreign institution?**

never/rarely/often/almost always/ I have never performed LIVE surgery in my home institution

**Has jet lag negatively affected your performance during LIVE surgery in the past?**

never/rarely/often/almost always

**When performing LIVE surgery, have you ever felt unfamiliar with the theater, staff or equipment, which negatively affected your performance?**

never/rarely/often/almost always

**When performing SEMI-LIVE surgery, do you prefer to record the surgical demonstrations yourself or would you rather have an external camera team over to record selected cases at a certain moment?**

I prefer to record and edit the demonstrations myself/ I prefer to have the camera team visit me to record the cases
